# Plant species occurrence patterns in Eurasian grasslands reflect adaptation to nutrient ratios

**DOI:** 10.1007/s00442-018-4086-6

**Published:** 2018-02-15

**Authors:** Ineke S. Roeling, Wim A. Ozinga, Jerry van Dijk, Maarten B. Eppinga, Martin J. Wassen

**Affiliations:** 1 Environmental Sciences, Copernicus Institute of Sustainable Development, Utrecht University, Heidelberglaan 2, PO Box 80115, 3508 TC Utrecht, The Netherlands; 2 Team Vegetation, Forest and Landscape Ecology, Wageningen Environmental Research (Alterra), Wageningen UR, PO Box 47, 6700 AA Wageningen, The Netherlands; 3 Institute for Water and Wetland Research, Radboud University Nijmegen, 6500 GL Nijmegen, The Netherlands

**Keywords:** Nitrogen, Phosphorus, Stoichiometry, Species composition, Niche

## Abstract

Previous studies of Eurasian grasslands have suggested that nutrient ratios, rather than absolute nutrient availabilities and associated productivity, may be driving plant species richness patterns. However, the underlying assumption that species occupy distinct niches along nutrient ratio gradients remains to be tested. We analysed plant community composition and nutrient status of 644 Eurasian wet grassland plots. The importance of nutrient ratios driving variation in species composition was analysed using ordination methods (DCA and CCA). Subsequently, we assessed the niche position and width along the most important nutrient ratio gradient [N:P] for each species. We found that the N:P ratio explained part of the variation in species composition independent from conventional explanatory variables. The N:P ratio explained less variation than soil moisture or pH, but more than productivity or the availability of N and P separately, highlighting its importance for grassland species composition. Species occupied distinct niches along the N:P gradient, and species’ niche widths decreased toward extreme nutrient limitation. After correcting for niche position, there was no overall difference in niche width between endangered and non-endangered species. Surprisingly, endangered species with niche optima at the extreme P-limited end of the gradient had broader niches than their non-endangered counterparts. As species occupied distinct niches along a nutrient ratio gradient, future grassland conservation efforts may benefit from targeting changes in nutrient ratios, i.e. the balance between N and P, rather than only focussing on a general reduction in nutrient availability. However, what management interventions can be used for this purpose remains unclear.

**Electronic supplementary material:**

The online version of this article (10.1007/s00442-018-4086-6) contains supplementary material, which is available to authorized users.

## Introduction

In recent decades, plant species richness of European (semi)natural grasslands has declined in response to various forms of anthropogenic pressure, including climate change and nutrient enrichment (Tamis et al. 2005; Dupré et al. 2010). As a result of this pressure, species composition in many types of grassland has changed, with common species increasing in abundance at the expense of rare species (e.g. Tamis et al. 2005). These observations have raised interest in identification of the mechanisms driving these changes (Hautier et al. 2009).

One mechanism that has received considerable attention is increased productivity in response to eutrophication (Gough et al. 2000; Crawley et al. 2005; Harpole and Tilman 2007). In highly productive grasslands, a few tall and fast-growing species outcompete slower growing species for light (Grime 2001; Hautier et al. 2009). This mechanism is supported by observations of simultaneous increases of grassland productivity and decreases in species richness in response to nutrient enrichment (Crawley et al. 2005, Hautier et al. 2009). More specifically, eutrophication has been linked to declining trends for species characteristic of nutrient-poor grasslands and increasing trends for species characteristic of nutrient-rich grasslands (Bobbink et al. 1998; Tamis et al. 2005).

Abiding by these findings, grassland conservation and restoration measures typically aim to reduce the availability of the main growth-limiting nutrients nitrogen (N), phosphorus (P) and potassium (K) to decrease site productivity (Olff and Bakker 1991; Berendse et al. 1992; Klimkowska et al. 2010). Until now, however, this strategy to reverse negative trends in grassland diversity has only yielded limited success (Berendse et al. 1992; Bakker and Berendse 1999; Van Dijk et al. 2007). This lack of success could be explained by the notion that nutrient supply ratios, rather than absolute amounts, are an additional important factor governing competition dynamics and species richness (Tilman 1982), apart from dispersal limitation (Ozinga et al. 2005). In this context, the ratio between N and P may be a particularly important driver (Braakhekke and Hooftman 1999; Cardinale et al. 2009).

In Eurasian grasslands and wetlands, species richness is highest at intermediate N:P ratios (Braakhekke and Hooftman 1999; Olde Venterink et al. 2003; Wassen et al. 2005; Fujita et al. 2014a), while the number and proportion of endangered species is higher at high N:P ratios (Wassen et al. 2005; Fujita et al. 2014a). Furthermore, considerable differences between N-limited and P-limited plant communities regarding their mean life-history traits have been observed along N:P gradients (Fujita et al. 2014a). Because plant species have different strategies regarding nutrient uptake, loss and recycling, their fitness is dependent on the relative availability of different nutrients (Olde Venterink and Güsewell 2010; Fujita et al. 2010a). This is in line with greenhouse experiments showing that the trait expression of individual species changes in response to the prevailing N:P ratio (Güsewell 2005a, b; Fujita et al. 2010a, b) and that this plasticity also affects the outcome of interspecific competition (Güsewell and Bollens 2003; Olde Venterink and Güsewell 2010). N:P ratios may therefore be regarded as a separate factor demarcating a species’ niche (sensu Chase and Leibold 2003), implying that an N:P gradient constitutes a niche gradient that may be driving at least part of the community composition. If this is the case, we would expect community composition to systematically change along N:P gradients, with each plant species occupying a clearly demarcated range of the N:P gradient.

In general, adaptation to extreme conditions might trade-off with trait plasticity (Sultan 2000; Fujita et al. 2014a), which implies that species adapted to extreme conditions should occupy smaller niches. It can therefore be hypothesized that niche widths along an N:P gradient become smaller towards lower and higher ends of the gradient (i.e. towards extreme N- and extreme P-limitation respectively). This notion becomes relevant when assessing species responses under changing environmental conditions, as species with the smallest niche widths will likely be the first to experience conditions beyond their niche. In a eutrophying world (sensu Steffen et al. 2015), the availability of both N and P may be increasing simultaneously. In systems characterized by extreme (i.e. either very low or very high) N:P ratios, simultaneous eutrophication with both N and P will proportionally increase the limiting nutrient the most. As a result, eutrophication processes may decrease the number of ecosystems characterized by extreme N:P ratios. Following from these two notions, it can be hypothesized that endangered species are characterized by having a relatively narrow niche at the extreme ends of the N:P gradient.

Previous studies have focussed either on changes in species composition at a local scale (Tilman 1982; Hegg et al. 1992; Roem and Berendse 2000; Gałka et al. 2005; Hejcman et al. 2007; Honsová et al. 2007; Chytrý et al. 2009), or on measures of species richness at larger spatial scales (Wassen et al. 2005; Fujita et al. 2014a). In this study, we focus on changes in species composition over large spatial scales, specifically testing the following hypotheses: (1) Nutrient ratio gradients explain part of the variation in species composition independent from other environmental factors. (2) Species occupy distinct niches along nutrient ratio gradients. (3) There is a general trade-off between a species’ niche position along a nutrient ratio gradient and its niche width. (4) Species currently endangered occur mainly under extreme nutrient limitation, and are more vulnerable to changes in nutrient supply due to their narrower niches along the nutrient ratio gradient. The aim of this study is to test these four hypotheses using a database containing information on plant species composition, productivity and aboveground tissue concentrations of N, P and K in herbaceous ecosystems across Eurasia (Fujita et al. 2014b).

## Materials and methods

### Compilation of the dataset

Starting point for our data analysis was the database collected by Fujita et al. (2014b; available through the Try Database: www.try-db.org/TryWeb/Data.php), comprising 690 plots within herbaceous ecosystems across Eurasia of moist grasslands including fens, bogs, marshes, reed beds, floodplains and dune slacks. The database contains the vascular plant species composition (presence/absence), aboveground standing biomass, N, P and K contents of the aboveground (living) biomass, and mowing frequency. Often, abundance data is preferred over presence/absence data (Ozinga et al. 2005). However, as a large part of the database lacked abundance data and we focus on large scale gradients, we decided to use the full dataset. To obtain a proxy for site productivity, aboveground standing biomass was harvested at the peak of the growing season, i.e. within the summer months (June, July or August). When site management included mowing, biomass was harvested before mowing took place. Mowing frequency was included because mowing combined with biomass removal is a known measure to reduce productivity and nutrient availability (Berendse et al. 1992; Bakker et al. 2002). In addition, mowing reduces light competition and can therefore directly impact species composition (Zobel 1992). In our dataset, mowing frequency ranged from zero to three times a year. Plots were excluded from the analysis if (a) more than 50% of the plot surface was covered with woody species; (b) K content of aboveground biomass was not measured, or (c) if the plots contained plants typical of saline (coastal) soils. Tree species, and species of which the identity was not unambiguously determined were removed from the dataset. The remaining 644 plots spanned eight Eurasian countries: the Netherlands (270 plots), Poland (153 plots), Germany (90 plots), Russia (82 plots), Belgium (20 plots), the United Kingdom (10 plots), Sweden (10 plots) and Belarus (9 plots). The final dataset contained 598 plant species, 229 of which are endangered (see below). Species richness varied between 2 and 66 species per plot. Plot sizes differed from 1–25 m^2^, but this has previously been shown not to affect relations between species richness and N:P stoichiometry (Fujita et al. 2014a). Plot productivity ranged from 30 to 2756 g/m^2^. In this study, we considered species to be ‘endangered’ when they were classified as ‘critically endangered’, ‘endangered’, or ‘vulnerable’ in either one or more of the Red Lists of the Netherlands, Germany, Poland, Sweden, the United Kingdom, and the Novosibirsk region in Siberia (following Fujita et al. 2014a).

### Nutrient ratios

Plant nutrient concentrations were previously measured in the aboveground biomass on plot level (Fujita et al. 2014a, b). Following earlier studies (Wassen et al. 1995, 2005, Olde Venterink et al. 2003; Van Bodegom et al. 2006; Eppinga et al. 2008, 2010), we use these plant nutrient concentrations—referred to in this paper as plant N, plant P and plant K—as a measure for plant available nutrient concentrations (see also Online Resource Appendix 1 for a further discussion on the reliability of plant nutrient concentrations relative to soil measurements).

Plant nutrient concentrations alone are not very useful as indicators of nutrient limitation, as a nutrient can be limiting even when it is available in high concentrations (Güsewell and Koerselman 2002). Nutrient ratios, on the other hand, are more reliable indicators as they correspond with the relative availability of N, P and K. The degree of K-limitation can be assessed using the N:K and K:P ratios, with K limiting biomass production when N:K > 2.1 and K:P < 3.4 (Olde Venterink et al. 2003). K-limitation is rare (Verhoeven and Schmitz 1991; Wassen et al. 2005) but for completeness these ratios were included. The N:P ratio expresses a gradient with varying degrees of N and P deficiency for plant growth and can be used to assess whether the production of biomass in a community is N-limited (N:P < 13.5), P-limited (N:P > 16) or co-limited (N:P 13.5–16; Güsewell and Koerselman 2002). The selected plots covered all three types of nutrient limitation, as the N:P ratios in plots ranged from 3.1 to 52.9. The usefulness and reliability of these ratios and their critical values has been established via field fertilisation experiments (Koerselman and Meuleman 1996; Verhoeven et al. 1996; Olde Venterink et al. 2003). Furthermore, N:P ratios differed more between sites than between species within a site, indicating that the N:P ratio reflects site nutrient availability (Güsewell and Koerselman 2002; Fujita et al. 2014a).

### Other environmental variables: Ellenberg indicator values

The dataset was further supplemented with environmental variables of known relevance for grassland species richness and community composition: soil moisture, soil pH, soil salinity, average annual temperature and light availability (Gough et al. 1994; Grace 1999; Cornwell and Grubb 2001; Grime 2001; Bobbink et al. 2010; Dupré et al. 2010). As these environmental factors were not measured, we estimated these factors using community averaged Ellenberg indicator values (EIVs—*Zeigerwerte*, Ellenberg et al. 1992). EIVs are commonly used in approaches describing environmental conditions experienced by plant communities (e.g. Cornwell and Grubb 2001; Lepik et al. 2005; Ozinga et al. 2005; Bernhardt-Römermann et al. 2008; Verheyen et al. 2008; Pruchniewicz 2017; Santini et al. 2017). These EIVs indicate, generally on an ordinal nine-point scale, under which field conditions a species is most likely to be found. The species physiological range is typically larger than indicated by the EIV, as the latter is based on field observations involving interspecific competition (Ellenberg et al. 1992). EIVs therefore indicate ecological optima and can be seen as analogue to realized niche optima (Cornwell and Grubb 2001). Following Hill and Carey (1997) and Ozinga et al. (2005), the plot environmental conditions at a given site were calculated as the average of the EIVs of all species present within the target plot.

### Effect of nutrient ratios on species composition

To test our first hypothesis, we used two complementary multivariate analyses to assess the importance of nutrient ratios for grassland species composition, relative to the absolute availability of each nutrient separately and to other environmental factors. First, a detrended correspondence analysis (DCA) was carried out to explore the main gradients in species composition and to check whether there was no major gradient in species composition that is not related to the environmental variables included in our study (Hill and Gauch 1980, Jongman et al. 1995). Nutrient ratios and other environmental variables were added as supplementary variables, making it possible to quantify the correlative strengths between these variables and the DCA axes (Table 1). We subsequently performed a canonical correspondence analysis (CCA) to quantify the relative importance of these variables in explaining variation in species composition.

**Table 1:** Summary of the detrended correspondence analysis (DCA) ordination showing overall variation in species composition and the important environmental variables

Ordination axis	Axis 1	Axis 2	Axis 3	Axis 4
Eigenvalues	0.71	0.37	0.25	0.19
Explained variation (cumulative %)	6.44	9.78	12.08	13.80
Gradient length	7.09	5.68	4.58	4.35
Species–environment correlations	0.96	0.86	0.79	0.71
Correlations of environmental variables with axes				
Moisture Ellenberg	0.91^b^	0.09	0.31	0.25
pH Ellenberg	− 0.59^a^	− 0.25	0.31	− 0.17
Mowing frequency	− 0.40^a^	− 0.27	− 0.23	− 0.34
N:P ratio plants	− 0.10	0.73^b^	0.27	0.44^a^
Plant P	− 0.06	− 0.62^b^	− 0.39	− 0.41^a^
Biomass production	0.08	− 0.48^a^	0.09	− 0.36
Temperature Ellenberg	0.29	− 0.20	0.60^b^	0.11
Salinity Ellenberg	− 0.15	− 0.49^a^	− 0.41^a^	− 0.37
Light Ellenberg	0.46^a^	0.49^a^	− 0.05	0.63^b^
K:P ratio plants	0.11	0.30	0.24	0.47
Plant N	− 0.20	0.02	− 0.06	− 0.12
N:K ratio plants	− 0.11	0.15	− 0.11	− 0.14
Plant K	0.06	− 0.30	0.01	0.15

Environmental variables included in the ordination are: Plant N; Plant P; Plant K; N:P ratio; N:K ratio; K:P ratio; Mowing frequencies and Ellenberg indicator values for soil moisture, soil pH, light, salinity and temperature. Environmental variables (used as supplementary variables) explained 21.1% of variation in species composition (adjusted explained variation is 19.5%). Correlation coefficients ≥ 0.4 or ≤ − 0.4 are indicated by superscript a, correlation coefficients ≥ 0.6 or ≤ − 0.6 by superscript b. The gradient length is a measure for the beta diversity in community composition (i.e. the extent of species turnover) along the individual ordination axes. The species–environment correlations measure the strength of the relation between response and explanatory variables for a particular ordination axis

For each evaluated variable we quantified its marginal (simple) effect and its conditional (partial) effect. The marginal effects were based on a model with only the target variable selected. For the quantification of the conditional effects, we performed for each target variable a partial CCA with all other variables (except the target variable) as covariates, thereby partialling out the shared effect of these variables on species composition. The conditional effect of the selected variable was tested by a partial Monte Carlo permutation test in which the residuals from the reduced model were permutated 1000 times. The reduced model thus included all other environmental variables that had already been selected, with the variable to be tested being excluded (Ter Braak and Šmilauer 2012). *p* values were adjusted for multiple testing using the false discovery rate.

In both the DCA and CCA, environmental variables were centred and standardized and rare species were down-weighted to minimize their influence on the outcomes. The multivariate analyses were executed using CANOCO 5 (Ter Braak and Šmilauer 2012).

### Species niche occupation along the N:P ratio gradient

To test our second hypothesis stating that species occupy distinct niches, we calculated the niche position along the N:P gradient for each species. As mentioned before, plant growth can also be limited by K but these occasions are rare (Güsewell and Koerselman 2002, Olde Venterink et al. 2003). Similarly, the results of the DCA and CCA analysis suggested that N:K and K:P ratios were less important for species composition than the N:P ratio. Hence, we focussed on the N:P ratio in the remainder of the study. In line with previous observations (Güsewell and Koerselman 2002), we found that plot N:P ratios were log-normally distributed along the gradient (mean plot N:P 11.2 mg N mg P^−1^), which can be explained by the numerical difference in the range of N (co-) limitation (3.1–16) and P (co-) limitation (13.5–52.9) within the dataset. We therefore log-transformed plot N:P ratios in subsequent analyses of species’ niche position and niche width. For each species, the niche position was calculated as the mean log[N:P] of all plots in which it was present. Niche positions were calculated only for species which occurred in at least 10 plots. This approach yielded niche positions for a pool of 269 species, 90 of which are endangered.

We compared the distribution of species niche positions along the N:P gradient to a null hypothesis stating that there is no relationship between plot N:P and species occurrence. To formalize this null hypothesis, we generated 1000 bootstrap replicates in which each plot received the same number of species as in the real dataset, but these species were randomly selected (with replacement, e.g. Efron and Tibshirani 1997) using the previously selected species pool. More specifically, we used the total number of observations of species presence in the dataset (*n* = 12,361 for the 269 species of the selected species pool) in the assembly procedure. This ensured that frequencies of species occurrence in the bootstrap replicates were similar to those observed in the real dataset. For both the real dataset and each of the bootstrap replicates, we assessed whether the calculated niche positions followed a uniform or bimodal distribution. In cases where a converging solution could be found for both distributions, the best fit was determined using a log-likelihood ratio test (Frankland and Zumbo 2009). For this test, the log-likelihood ratio of the data (− 2ln(Λ_data_)) was calculated and compared with critical log-likelihood values of a theoretical unimodal sampling distribution (− 2ln(Λ)). In addition, the difference in variance explained by the bimodal fit and the unimodal fit to the data (Δ*R*^2^) was compared to critical values of a Δ*R*^2^ sampling distribution. Critical values were given for *p* = 0.10, 0.05 and 0.01. In this manner, qualitative differences in modality between the real dataset and bootstrap replicate fits could be identified. Quantitatively, we compared the distribution in the real dataset with 1000 realizations (of equal sample size) of the distribution generated with the bootstrapping procedure described above. Distributions were compared with a two-sample Kolmogorov–Smirnov test, and the results were summarized by the average test value, ${\bar{\text{D}}}_{269,269}$, and average probability of exceedance, $\bar{p}$, of the 1000 comparisons made. The analyses involving bootstrap replicates were carried out using MATLAB (v. 8.5; Mathworks, 2015), and log-likelihood ratio tests were carried out in SPSS (v. 22.0; IBM Corp., Armonk, NY, USA, 2013).

### Trade-offs between specialisation and niche width

The third hypothesis stated that there is a general trade-off between a species’ niche position along a nutrient ratio gradient and its niche width. Following Ozinga et al. (2013), we defined the niche width of a species as two times the standard deviation of the log[N:P] values of the vegetation of all plots in which the species occurred. Subsequently, species’ niche widths were plotted against niche positions. As the variances were heterogeneous, the relationship was analysed using quantile regressions (Cade and Noon 2003). A quadratic relationship was assumed, as we hypothesized that niche widths would be smaller towards more extreme N- and P-limitation. To establish the relationship between niche position and niche widths, the quadratic quantile regressions were calculated using the 0.50 (i.e. the median), 0.75, 0.90 and 0.95 quantiles (following Fujita et al. 2014a). Confidence intervals (95%) were calculated for both quadratic and linear regression terms. Again, we formalized a null hypothesis by following the above procedure for each of the 1000 bootstrap replicates described above. Using the 0.50 quantile regressions for each replicate, we could construct an average regression equation (and 95% confidence intervals) under the null hypothesis using a non-parametric procedure (e.g. Efron and Tibshirani 1997; Eppinga et al. 2010). These quantile regressions were executed in R (v. 3.3.3; The R Foundation for Statistical Computing, 2017). As we found a quadratic relationship for both the real dataset and for the bootstrap replicates generated under the null hypothesis, we performed an additional test comparing the magnitude of the decrease in niche width towards the extreme ends of the N:P gradient. For both the real dataset and the bootstrap replicates, we separated the dataset in an N-(co-)limited part and a P-(co-)limited part, and fitted for both subsets a linear relationship between niche position and niche width. For the 1000 bootstrap replicates, this procedure generated a probability distribution for the magnitude of the decrease in niche widths towards the extreme ends of the N:P gradient under the null hypothesis. This probability distribution could then be used to quantify the strength of the trends observed in the real dataset, again using the non-parametric procedure described above (e.g. Efron and Tibshirani 1997; Eppinga et al. 2010).

### Niche widths of endangered and non-endangered species

The fourth hypothesis stated that species currently endangered occur mainly under conditions of extreme nutrient limitation and therefore have a narrower niche. To test this hypothesis, we used the general relationship between niche position and niche width (the median quantile regression described above) to calculate residual niche widths. Here, a positive residual indicated a niche width larger than predicted. We first compared the residual niche widths for endangered and non-endangered species, hypothesizing that endangered species would have smaller residual niche widths. We used a Mann–Whitney *U* test (with exact significance), as the assumptions of (log−) normally distributed variables and homogeneity of variances were not met. We then tested whether there was a correlation between niche position extremity (quantified as absolute distance from the dataset’s mean log[N:P]) and residual niche widths for both non-endangered and endangered species by means of a non-parametric one-sided Spearman correlation test. These analyses were carried out using SPSS (v. 22.0; IBM Corp., Armonk, NY, USA, 2013).

## Results

### N:P ratio explains part of the variation in species composition

**Fig. 1:**
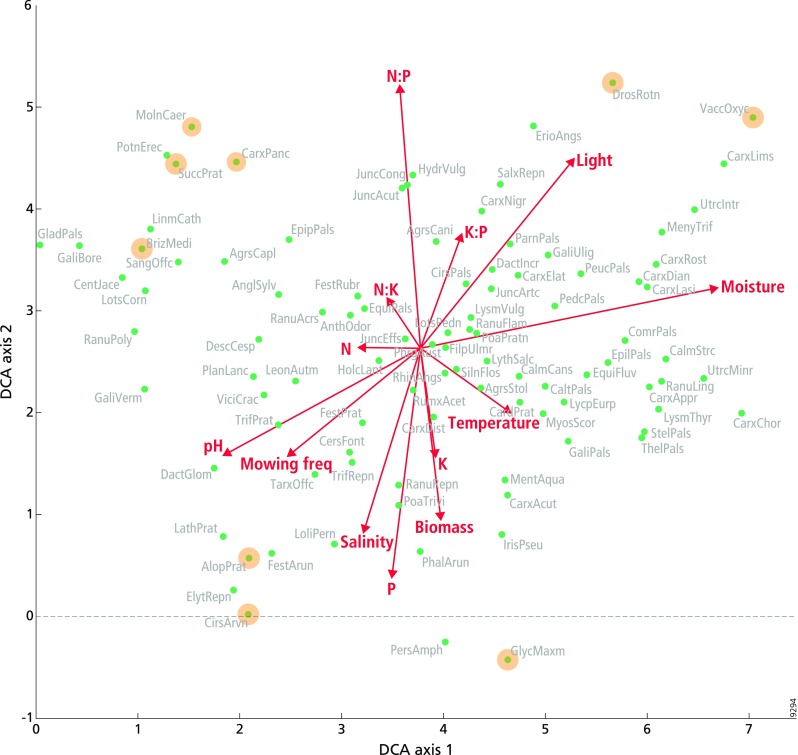
Detrended correspondence analysis (DCA) biplot, showing the relationship between environmental variables (red arrows) and species composition (green circles). Arrow length reflects the magnitude of the effect on species composition. The value of the variable increases along the arrow. Yellow circles indicate species specifically mentioned in the results section. Full species names are given in Online Resource Appendix 2 Table S1. For clarity, only the 98 most abundant species are represented

The DCA showed a clear relationship between the main gradients in species composition and some of the environmental variables included in the analysis (Fig. 1, Table 1). Moreover, it showed that there are no major gradients in species composition that are not related to environmental variables, which implies that the subsequent CCA does not miss important variability in species composition that is not related to the environmental variables. Figure 1 shows a soil moisture gradient along the horizontal (first) axis, with species growing under wetter conditions at the right side of the graph. The horizontal axis was also relatively strongly correlated with soil pH (Table 1). The vertical (second) axis was mainly determined by N:P ratio and plant P concentrations. This implies that plants occurring in the top half of the figure grow under lower P availability than those in the lower half, since the high N:P ratio is mainly driven by P availability. Under these conditions, smaller statured species characteristic for soils that are wet and acidic, such as *Drosera rotundifolia* and *Vaccinium oxycoccos* (upper right corner), can be found under conditions further characterized by high light availability. The upper left corner of the diagram is characterized by species associated with *Cirsio dissecti*-*Molinietum* grasslands (e.g. *Succisa pratensis, Molinia caerulea*, *Carex panicea* and *Briza media*), under conditions of low P availability, low soil moisture, high pH and intermediate mowing frequency. In contrast, larger statured species associated with productive grasslands, such as *Alopecurus pratensis*, *Cirsium arvense* and *Glyceria maxima*, are found in the lower half of the graph, which is characterized by a high productivity, a low N:P ratio and a high P availability, high salinity, high soil pH and high mowing frequency.

The CCA showed that all environmental variables together explained 21.1% of the variation in species composition. Table 2 gives an overview of the marginal (simple) effects and the conditional (partial) effects of each variable. The N:P ratio explained 3.7% of the variation in species composition (Table 2, marginal effects), which is comparable to the marginal effects of light availability and soil salinity (3.7 and 3.6%, respectively), but less than soil moisture and soil acidity and more than productivity (5.7, 4.1 and 1.6%, respectively). In comparison to N:P ratio, N and P availability alone explained less variation (N: 1.0%, P: 2.9%). When considering the conditional effects (Table 2), the N:P ratio explained 1.2% of the variation in species composition, which was a larger proportion than conditionally explained by any of the other nutrient-based variables. This result confirms that the effect of N:P ratio on species composition is partly independent of the effects of the absolute availability of nutrients and productivity.

**Table 2:** Summary of the canonical correspondence analysis (CCA), showing the marginal (simple) and conditional effects of the selected environmental variables on plant species composition

Marginal effects			Conditional effects		
Environmental variable	Explains %	Pseudo F	Environmental variable	Explains %	Pseudo F
Moisture Ellenberg	5.7	38.9***	Moisture Ellenberg	3.1	20.2***
pH Ellenberg	4.1	27.2***	pH Ellenberg	2.5	16.1***
N:P ratio plants	3.7	24.9***	Temperature Ellenberg	1.8	11.6***
Light Ellenberg	3.7	24.4***	Salinity Ellenberg	1.3	8.2***
Salinity Ellenberg	3.6	24.0***	N:P ratio plants	1.2	7.5***
Mowing frequency	3.0	19.9***	Light Ellenberg	1.2	7.7***
Plant P	2.9	19.1***	Mowing frequency	1.1	6.8***
K:P ratio plants	2.3	14.9***	Plant K	0.7	4.4***
Temperature Ellenberg	2.3	14.8***	K:P ratio plants	0.7	4.3***
Biomass production	1.6	10.7***	Plant N	0.7	4.7***
Plant K	1.1	7.0***	Plant P	0.6	3.5***
Plant N	1.0	6.7***	Biomass production	0.5	3.2***
N:K ratio plants	1.0	6.4***	N:K ratio plants	0.4	2.2***

Conditional effects are based on partial CCA with all variables except the target variable included as covariates, using partial Monte Carlo permutation tests. All selected environmental variables contribute significantly to the model (False discovery rate *p* < 0.001, ***)

### Species occupy distinct niches along the N:P ratio gradient

**Fig. 2:**
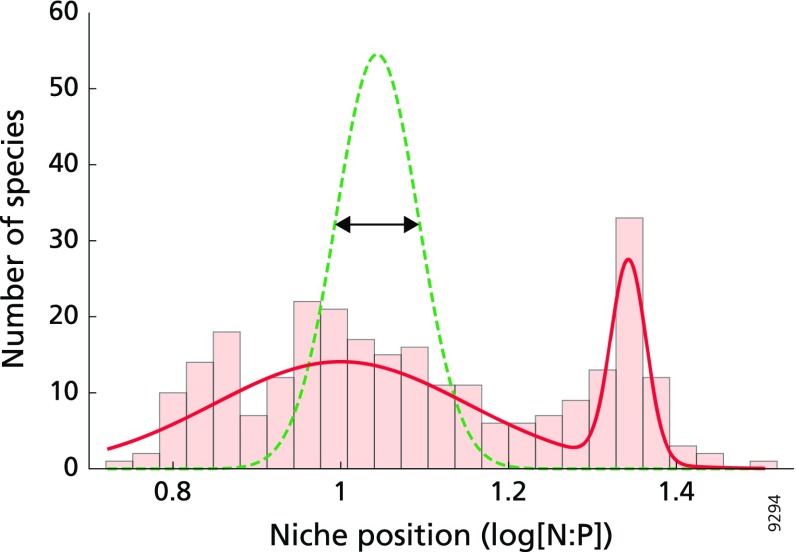
Histogram depicting the species distribution according to their niche position. The niche position is calculated at the log[N:P ratio] scale. The red solid line indicates the bimodal distribution of the dataset. The green dotted line indicates the unimodal distribution of 1000 bootstrapped dataset replicates; the arrow marks the ± 1 standard deviation around the bootstrapped mean. Conditions are N-limited when log[N:P] < 1.13; conditions are P-limited when log[N:P] > 1.20

Under the null hypothesis (green dotted line in Fig. 2, no association between species occurrence and N:P ratio), optimum niche positions were narrowly and unimodally distributed around the mean plot N:P ratio. In contrast, the actual niche positions revealed a different distribution (2-sample Kolmogorov–Smirnov test; ${\bar{\text{D}}}_{269,269}$ = 0.365, $\bar{p}$ < 0.001) in that species occupied distinct niches along the N:P gradient (bars and fitted solid line in Fig. 2). The distribution of niche positions was clearly bimodal (− 2ln(Λ_data_) = 98.05, *p* < 0.01; Δ*R*^2^ = 0.293, *p* < 0.01). This bimodality consisted of one mode encompassing species with a niche optimum under very high N:P ratios (Fig. 2). The second mode encompassed species with niche optima within the N-(co-)limited part of the gradient. Within this second mode, species were more uniformly distributed along a wider range of the N:P gradient (red solid line in Fig. 2).

### Niches are narrower at the extreme N:P ratios

**Fig. 3:**
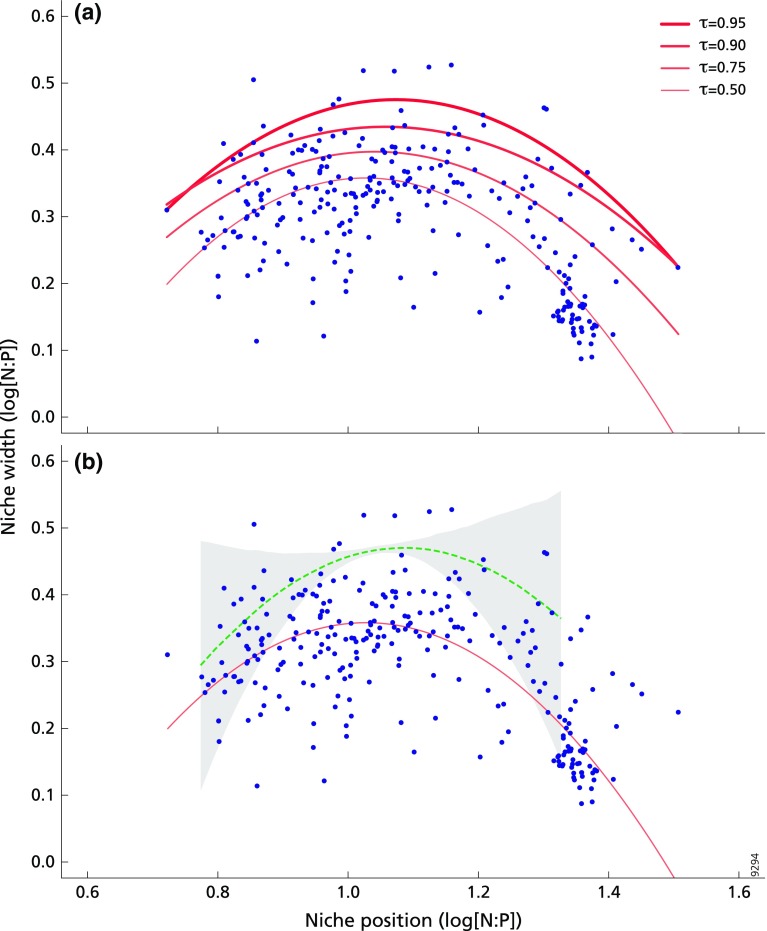
Relationship between niche position and niche width, both calculated using log[N:P]. Each blue dot represents one of 269 species. **a** The red lines depict the τth quantile regression functions (for *τ* = 0.50, 0.75, 0.90, 0.95). See Online Resource Appendix 3 Fig. S1 for the 95% confidence intervals of the regression coefficients. **b** The median quantile regression for niche position and niche width of the data (red line) in comparison to the median quantile regressions of 1000 random datasets (median indicated by the green dotted line, 95% confidence interval indicated by the grey area)

Niche widths were largest at intermediate N:P ratios, i.e. around the transition of N- to co-limitation (Fig. 3a), which was consistent across the different quantiles examined. This was also the case for the simulated data (Fig. 3b). It is noteworthy, however, that around intermediate niche positions the individual species show a large range of niche widths (varying from relatively small to broad, blue dots), whereas the niche widths of the simulated data were always broad (narrow 95% CI, grey area). In general, real species had smaller niche widths than simulated species (Fig. 3b). Furthermore, niche widths become smaller towards more extreme N:P ratios and this overall pattern is found for both the real dataset (red line, Fig. 3b) and the bootstrap replicates simulated under the null hypothesis (green dotted line). Comparing the strengths of these trends in the simulated and real dataset, we found that the decrease in niche width was not significantly different for N-limited systems (slope_real_ = − 0.15 slope_simulated_ = − 0.39, *p* = 0.12), but the decrease in niche width for P-limited systems was stronger than the decrease observed under the null hypothesis (slope_real_ = − 0.59 slope_simulated_ = − 0.09, *p* = 0.012).

### Endangered species do not necessarily have a narrow niche

**Fig. 4:**
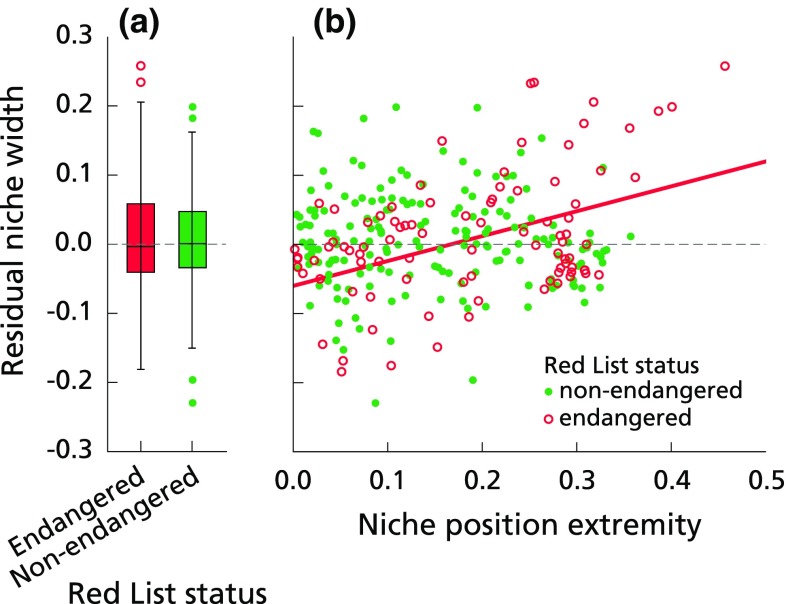
**a** Boxplots of the niche widths for non-endangered and endangered species. Error bars indicate ± 2 SE, *N* = 269. The residual niche width is calculated as the deviance between a species’ real niche width and the expected species niche width as predicted by the median quantile regression. Positive residuals indicate larger than expected niche widths. **b** The relationship between niche position extremity and residual niche width. Niche position extremity is calculated as the distance between niche position and the mean log[N:P] for all plots in the dataset; a higher value indicates more extreme N- or P-limitation. The expected niche width is depicted by the horizontal line at *y* = 0. The red line depicts the only significant relationship, which occurred for endangered species

Endangered species had slightly lower residual niche widths when compared to non-endangered species (median values − 0.0035 and 0.0007, respectively, Fig. 4a) but this difference was not statistically significant (one-sided Mann–Whitney *U* test, *U* = 8052.00, *p* = 0.498). Furthermore, residual niche widths of endangered species increased with niche position extremity (Fig. 4b. *r*_s_ = 0.313, *p* < 0.01). In other words, endangered species with niche optima at the extreme ends of the N:P ratio gradient maintained broader niches than their non-endangered counterparts. This was caused solely by a niche width increase of species with co/P-limited niche positions (*r*_s_ = 0.256, *p* < 0.05), as there was no relationship between niche width and niche position extremity for species with N-limited niche positions (*r*_s_ = 0.088, *p* = 0.368). There was no overall relationship between niche width and niche position extremities for non-endangered species (Fig. 4b; *r*_s_ = − 0.076, *p* = 0.157).

## Discussion

All environmental variables included in our CCA-model—i.e. NPK availability, NPK ratios, productivity, soil moisture, salinity, pH, temperature and light availability—combined explained more than 20% of the total variation in the species composition of the Eurasian grassland dataset. This is a relatively high proportion of variation explained for observational data with only information on species presences and absences, where dispersal limitation plays an important role at the spatial scales captured (Ozinga et al. 2005). We found that the N:P ratio explained a unique, and compared to other explanatory environmental variables, relatively large part of the variation in species composition among Eurasian grasslands (marginal effects, Table 2). When considering unique (i.e. conditional) effects, proportions of variation explained decrease due to correlations among environmental variables. Nevertheless, even in this more conservative approach, the N:P ratio explained similar proportions of variation in species composition as the more conventional determinants (Table 2). Interestingly, we also found that the N:P ratio conditionally explained more variation than other nutrient-based variables and productivity. At first glance, this seems to contradict with the recent meta-analysis of Soons et al. (2017), who found that eutrophication with N is responsible for species loss, whereas the influence of P is negligible. Their results indicate that high atmospheric N deposition rates lead to an increased biomass, which may lead to species loss. However, the authors themselves note that they have looked at species numbers, and suggest that addition of P might lead to ‘loss’ via species turnover, while simultaneously maintaining stable species numbers. Our study provides evidence for this suggestion and underlines the importance of including species turnover in addition to biodiversity metrics when studying the impact of eutrophication. It therefore seems that N and P indeed influence the ecosystem via different mechanisms (Soons et al. 2017) as could already be surmised by the differences in species richness-productivity curves for different limitation types (Wassen et al. 2005).

To our knowledge, our analysis of Eurasian grasslands is the first to clearly show that species occupy distinct niches along a N:P gradient (Fig. 2). Species’ optima occur along a much broader range of the N:P gradient than expected and the bimodal distribution suggests that species can be roughly classified in two groups: species with an optimum at P-limited conditions and species with an optimum at N-(co-)limited conditions (Fig. 2). In addition, our analysis also provides support for a trade-off between a plant species’ niche position along a nutrient ratio gradient and its niche width under P-limitation, but not under N-limitation (Fig. 3). This trade-off is in line with the finding that species which, according to their field distribution, appear to be (highly) specialised in growing under extreme nutrient limitation, cannot successfully grow and compete under a nutrient limitation type that strongly deviates from that optimum (Olde Venterink and Güsewell 2010; Fujita et al. 2010a). Species have specific trait-based strategies to cope with nutrient limitation (Fujita et al. 2010a). If one or more of these traits are not (sufficiently) plastic, it can harm the plants’ performance when nutrient conditions deviate from the species-specific optimum. For example, species that generally occur in P-limited grasslands had the highest (relative) phosphatase production in comparison to other species, which proved to be a useful strategy (Olde Venterink and Güsewell 2010; Fujita et al. 2010b). However, in a competition experiment, the P-limited specialist still exuded quite high amounts of phosphatase under N-limited conditions (Olde Venterink and Güsewell 2010), which might be a disadvantage in competition. An implication of this notion is that species loss due to eutrophication may be particularly likely when increased supply of nutrients shifts the balance towards a state of less extreme nutrient limitation or even to another nutrient limitation type.

Previous studies showed that P-limited plots contained more endangered species (Wassen et al. 2005; Fujita et al. 2014a) and it was suggested that traits enabling tolerance of P-limited conditions (such as conservative growth and reproductive strategies) also make these species more vulnerable for (local) extinction (Fujita et al. 2014a). We therefore hypothesized that endangered species were at risk because they had even narrower niches than their non-endangered counterparts. This, however, appeared not to be the case as there was no significant difference between the expected niche widths of endangered and non-endangered species (Fig. 4a). On the contrary, under conditions of P-limitation residual niche widths of endangered species increased with increasing nutrient limitation (Fig. 4b), which refutes our hypothesis. Some of the endangered species with a P-limited optimum had very large niche widths that spanned to the co-limited and even N-limited regions of the N:P gradient. Among these species are *Drosera rotundifolia*, *Calluna vulgaris*, *Vaccinium oxycoccos*, *Carex limosa*, *Platanthera bifolia* and *Briza media*. One potential explanation for this unexpected pattern is that some of these co-limited or N-limited sites used to be P-limited, but eutrophication has significantly altered the balance between N and P in these sites. This seems a reasonable hypothesis as human impact has drastically increased the input of P to ecosystems around the globe (Steffen et al. 2015), and there is evidence that the P cycle is more disrupted than the N cycle (Falkowski et al. 2000). This may have resulted in a shift from P-limitation to N-limitation in many areas (Wassen et al. 2005). It has even been stated that the critical effect of P on species richness has been overlooked by many scientists and needs more attention in ecology (Ceulemans et al. 2012; Fay et al. 2015). All of the above mentioned species occur in sites with low productivity and a low availability of P (Fig. 1), implying that small changes in nutrient availability in these sites may lead to large changes in N:P ratio. Endangered species may be in the process of getting excluded from these sites, even though their current presence includes those sites as part of its niche. The extinction debt, the delayed extinction as a result of habitat modification or loss, is higher for endangered, specialised, long-lived species with low dispersal capabilities (Kuussaari et al. 2009). Fujita et al. (2014a) found that P-limited communities consisted of longer lived species that invested less in seeds as compared to N-limited communities, making it likely that endangered species with a niche optimum at P-limited conditions contribute disproportionately to the extinction debts of (semi-)natural grasslands. The possibility of species exclusion can be tested by calculating whether species abundance is significantly increasing with increasing plot N:P ratios, i.e. if species abundance is higher at P-limited plots compared to N- and co-limited plots. Unfortunately, a large part of our database lacks abundance data, and the subset that is available is too small to thoroughly test this hypothesis. Nevertheless, our methodology based on species presence therefore likely overestimates the niche width for these species in the current approach. Such historical effects could be assessed in future efforts by monitoring trends in species abundance across the N:P gradients in which they are present. Although these trends will be highly variable and affected by many other factors, including interspecific competition, species for which many populations occur outside their N:P niche are likely to show consistent negative population trends. Another potential explanation is that these species occur under specific environmental conditions, of which the N:P ratio is only one—less limiting—dimension. *Drosera rotundifolia* and *Carex limosa*, for example, are species that occur in open vegetation, growing on wet and acid soils (Fig. 1). It is likely that their niches for soil moisture and acidity will be narrow.

Management actions aimed at combating negative trends in species diversity have mainly focussed on reducing the total amount of one or more of the plant available macronutrients N, P and K (Koerselman et al. 1990; Berendse et al. 1992; Olff et al. 1994; Jansen and Roelofs 1996; Patzelt et al. 2001; Hölzel and Otte 2003; Rasran et al. 2007), rather than targeting the nutrient supply ratio (Verhoeven and Schmitz 1991; Bakker and Olff 1995; Olde Venterink et al. 2009). Our results, however, suggest that the latter is at least as important for species composition as the former, and that restoration measures that take the stoichiometric requirements of the target vegetation into account, may therefore be more effective in restoring grassland diversity. We note that data on historic stoichiometric balances is scarce for most restoration sites. However, when historic species composition data is available, our results can provide another way forward to inform restoration management. Given that our approach enables an inference of species optima and species niches along nutrient ratio gradients, this information could be used to make an assessment of the preferred community average N:P supply ratio of the historic vegetation types (based on niche preferences of the historic species set). Discrepancies between those ratios and the modern N:P supply ratio (inferred from current plant tissue N and P) can then be used to inform potential management actions. This approach can even be used in the absence of historic vegetation data, as long as clear vegetation targets have been defined. However, steering on stoichiometry remains difficult. Currently, no specific management options targeting stoichiometry exist, and the few studies that have addressed the effects of management interventions on site N:P ratios showed conflicting results (e.g. Verhoeven and Schmitz 1991; Bakker & Olff 1995; Olde Venterink et al. 2009).

To conclude, by linking nutrient supply ratios to species niches, our findings may enable the generation of more specific hypotheses regarding the role of nutrient stoichiometry in species assembly processes, as well as plant community dynamics in response to global change. Furthermore, the relative importance of N:P ratios for community composition compared to other environmental factors that we demonstrated here warrants more research into management interventions that target specific nutrients (and thereby nutrient ratios) to improve the effectiveness of conservation management and restoration efforts.

## Electronic supplementary material

Below is the link to the electronic supplementary material.

Supplementary material 1 (PDF 255 kb)
