# Supplementary material for: Plant species occurrence patterns in Eurasian grasslands reflect adaptation to nutrient ratios
Source: Oecologia. 2018 Feb 15;186(4):1055–67. doi: 10.1007/s00442-018-4086-6 (PMC5859057; doi:10.1007/s00442-018-4086-6)
Supplement: Supplementary file 1 — Supplementary material 1 (PDF 255 kb) [file 442_2018_4086_MOESM1_ESM.pdf]

Electronic supplementary material

Plant species occurrence patterns in Eurasian grasslands reflect adaptation to nutrient ratios

Roeling, I.S.[1,*], Ozinga, W.A.[2,3], van Dijk, J.[1], Eppinga, M.B.[1] and Wassen, M.J.[1] 2017.

[1] Environmental Sciences, Copernicus Institute of Sustainable Development, Utrecht University, Heidelberglaan 2, PO Box 80115, 3508 TC Utrecht, The Netherlands

[2] Team Vegetation, Forest and Landscape Ecology, Wageningen Environmental Research (Alterra), Wageningen UR, PO Box 47, 6700 AA Wageningen, The Netherlands and

[3] Institute for Water and Wetland Research, Radboud University Nijmegen, 6500 GL Nijmegen, The Netherlands

[*]Corresponding author: I.S.Roeling@uu.nl

Appendix 1. Using plant nutrient concentrations as indicators of plant nutrient availability

The database used in this study (Fujita et al. 2014b) contained nutrient concentrations measured in the aboveground biomass (AGB). The values were measured by taking a subsample of the AGB for every plot, after which all subsamples were dried and chemically analysed for N, P and K concentrations (Fujita et al. 2014a). These nutrient concentrations – plant N, plant P and plant K – give a good indication of the plant available nutrient concentrations. Nutrient concentrations measured in the AGB do not necessarily correspond with nutrient concentrations in the soil (Güsewell and Koerselman 2002), the latter being often used as plant available nutrient concentrations. However, AGB nutrient concentrations potentially provide a better indication of plant available nutrient concentrations. First, nutrient concentrations often show strong temporal variation and a soil sample only provides a 'snapshot' of the soil nutrient concentrations at the time of sampling (Wheeler et al. 1992; Güsewell and Koerselman 2002). Nutrient concentrations measured in the AGB, on the other hand, are assumed to provide an overview of the nutrient uptake during the growing season (Vermeer and Berendse 1983; Güsewell and Koerselman 2002). This argument is supported by the fact that an increased nutrient availability also leads to an increased nutrient concentration in aboveground biomass, as both field and greenhouse fertilisation experiments have shown (Solander 1983; Verhoeven and Schmitz 1991; Craft et al. 1995; Güsewell and Koerselman 2002). Second, there are many different methods to extract nutrients from the soil and which methodology is most suitable to determine nutrient availability as perceived by plants is still being debated and investigated (Gilbert et al. 2009; Wuenscher et al. 2015). Third, it is also likely that soil nutrient measurements underestimate the amount of plant available nutrients, as plants have developed all kinds of mechanisms to take up 'unavailable' nutrients such as the production of phosphatase to access bound-phosphorus (Pérez Corona et al. 1996; Fujita et al. 2010), the direct uptake of amino acids (Streeter et al. 2000; Lipson and Näsholm 2001; Weigelt et al. 2005) and symbiotic N2 fixation (Aerts and Chapin III 1999). Mechanisms like these may be especially important in a study such as ours, where 516 out of 544 plots have a low biomass production (<600g/m2) and where competition for available nutrients might be fierce.

References

Aerts R, Chapin III FS (1999) The Mineral Nutrition of Wild Plants Revisited: A Re-evaluation of Processes and Patterns. In: Advances in Ecological Research. pp 1–67

Craft CB, Vyzamal J, Richardson CJ (1995) Response of Everglades plant communities to nitrogen and phosporus additions. Wetlands 15:258–271. doi: 10.1007/BF03160706

Fujita Y, Robroek BJM, De Ruiter PC, Heil GW, Wassen MJ (2010) Increased N affects P uptake of eight grassland species: the role of root surface phosphatase activity. Oikos 119:1665–1673. doi: 10.1111/j.1600-0706.2010.18427.x

Fujita Y, Venterink HO, van Bodegom PM, Douma JC, Heil GW, Hölzel N, Jabłońska E, Kotowski W, Okruszko T, Pawlikowski P, de Ruiter PC, Wassen MJ (2014b) Data from: Low investment in sexual reproduction threatens plants adapted to phosphorus limitation. Try Database. www.try-db.org/TryWeb/Data.php.

Fujita Y, Venterink HO, van Bodegom PM, Douma JC, Heil GW, Hölzel N, Jabłońska E, Kotowski W, Okruszko T, Pawlikowski P, de Ruiter PC, Wassen MJ (2014a) Low investment in sexual reproduction threatens plants adapted to phosphorus limitation.

Nature 505:82–6. doi: 10.1038/nature12733
Gilbert J, Gowing D, Wallace H (2009) Available soil phosphorus in semi-natural grasslands: Assessment methods and community tolerances. Biol Conserv 142:1074–1083. doi: 10.1016/j.biocon.2009.01.018
Güsewell S, Koerselman W (2002) Variation in nitrogen and phosphorus concentrations of wetland plants. Perspect Plant Ecol Evol Syst 5:37–61. doi: 10.1078/1433-8319-0000022
Lipson D, Näsholm T (2001) The unexpected versatility of plants: organic nitrogen use and availability in terrestrial ecosystems. Oecologia 128:305–316. doi: 10.1007/s004420100693
Pérez Corona ME, van der Klundert I, Verhoeven JTA (1996) Availability of organic and inorganic phosphorus compounds as phosphorus sources for Carex species. New Phytol 133:225–231. doi: 10.1111/j.1469-8137.1996.tb01889.x
Solander D (1983) Biomass and shoot production of Carex rostrata and Equisetum fluviatile in unfertilized and fertilized subarctic lakes. Aquat Bot 15:349–366. doi: 10.1016/0304-3770(83)90003-7
Streeter TC, Bol R, Bardgett RD (2000) Amino acids as a nitrogen source in temperate upland grasslands: the use of dual labelled (13 C, 15 N) glycine to test for direct uptake by dominant grasses. Rapid Commun Mass Spectrom 14:1351–1355.
Verhoeven JTA, Schmitz MB (1991) Control of plant growth by nitrogen and phosphorus in mesotrophic fens. Biogeochemistry 12:135–148. doi: 10.1007/BF00001811
Vermeer JG, Berendse F (1983) The relationship between nutrient availability, shoot biomass and species richness in grassland and wetland communities. Vegetatio 53:121–126. doi: 10.1007/BF00043032
Weigelt A, Bol R, Bardgett RD (2005) Preferential uptake of soil nitrogen forms by grassland plant species. Oecologia 142:627–635. doi: 10.1007/s00442-004-1765-2
Wheeler BD, Shaw SC, Cook RED (1992) Phytometric Assessment of the Fertility of Undrained Rich-Fen Soils. J Appl Ecol 29:466–475. doi: 10.2307/2404514
Wuenscher R, Unterfrauner H, Peticzka R, Zehetner F (2015) A comparison of 14 soil phosphorus extraction methods applied to 50 agricultural soils from Central Europe. 61:86–96. doi: 10.17221/932/2014-PSE

Appendix 2. Acronyms and accompanying species names

Table S1. Acronyms and accompanying species names, as depicted in the DCA biplot (Figure 1).

**Acronym**	**Species name**
AgrsCani	*Agrostis canina*
AgrsCapl	*Agrostis capillaris*
AgrsStol	*Agrostis stolonifera*
AlopPrat	*Alopecurus pratensis*
AnglSylv	*Angelica sylvestris*
AnthOdor	*Anthoxanthum odoratum*
BrizMedi	*Briza media*
CalmCans	*Calamagrostis canescens*
CalmStrc	*Calamagrostis stricta*
CaltPals	*Caltha palustris*
CardPrat	*Cardamine pratensis*
CarxAcut	*Carex acuta*
CarxAppr	*Carex appropinquata*
CarxChor	*Carex chordorrhiza*
CarxDian	*Carex diandra*
CarxDist	*Carex disticha*
CarxElat	*Carex elata*
CarxLasi	*Carex lasiocarpa*
CarxLims	*Carex limosa*
CarxNigr	*Carex nigra*
CarxPanc	*Carex panicea*
CarxRost	*Carex rostrata*
CentJace	*Centaurea jacea*
CersFont	*Cerastium fontanum*
CirsArvn	*Cirsium arvense*
CirsPals	*Cirsium palustre*
ComrPals	*Comarum palustre*
DactGlom	*Dactylis glomerata*
DactIncr	*Dactylorhiza incarnata*
DescCesp	*Deschampsia cespitosa*
DrosRotn	*Drosera rotundifolia*
ElytRepn	*Elytrigia repens*
EpilPals	*Epilobium palustre*
EpipPals	*Epipactis palustris*
EquiFluv	*Equisetum fluviatile*
EquiPals	*Equisetum palustre*
ErioAngs	*Eriophorum angustifolium*

FestArun	*Festuca arundinacea*
FestPrat	*Festuca pratensis*
FestRubr	*Festuca rubra*
FilpUlmr	*Filipendula ulmaria*
GaliBore	*Galium boreale*
GaliPals	*Galium palustre*
GaliUlig	*Galium uliginosum*
GaliVerm	*Galium verum*
GladPals	*Gladiolus palustris*
GlycMaxm	*Glyceria maxima*
HolcLant	*Holcus lanatus*
HydrVulg	*Hydrocotyle vulgaris*
IrisPseu	*Iris pseudacorus*
JuncAcut	*Juncus acutiflorus*
JuncArtc	*Juncus articulatus*
JuncCong	*Juncus conglomeratus*
JuncEffs	*Juncus effusus*
LathPrat	*Lathyrus pratensis*
LeonAutm	*Leontodon autumnalis*
LinmCath	*Linum catharticum*
LoliPern	*Lolium perenne*
LotsCorn	*Lotus corniculatus*
LotsPedn	*Lotus pedunculatus*
LycpEurp	*Lycopus europaeus*
LysmThyr	*Lysimachia thyrsiflora*
LysmVulg	*Lysimachia vulgaris*
LythSalc	*Lythrum salicaria*
MentAqua	*Mentha aquatica*
MenyTrif	*Menyanthes trifoliata*
MolnCaer	*Molinia caerulea*
MyosScor	*Myosotis scorpioides*
ParnPals	*Parnassia palustris*
PedcPals	*Pedicularis palustris*
PersAmph	*Persicaria amphibia*
PeucPals	*Peucedanum palustre*
PhalArun	*Phalaris arundinacea*
PhrgAust	*Phragmites australis*
PlanLanc	*Plantago lanceolata*
PoaPratn	*Poa pratensis*
PoaTrivi	*Poa trivialis*
PotnErec	*Potentilla erecta*
RanuAcrs	*Ranunculus acris*
RanuFlam	*Ranunculus flammula*
RanuLing	*Ranunculus lingua*

RanuPoly	*Ranunculus polyanthemos*
RanuRepn	*Ranunculus repens*
RhinAngs	*Rhinanthus angustifolius*
RumxAcet	*Rumex acetosa*
SalxRepn	*Salix repens*
SangOffc	*Sanguisorba officinalis*
SilnFlos	*Silene flos-cuculi*
StelPals	*Stellaria palustris*
SuccPrat	*Succisa pratensis*
TarxOffc	*Taraxacum officinale*
ThelPals	*Thelypteris palustris*
TrifPrat	*Trifolium pratense*
TrifRepn	*Trifolium repens*
UtrcIntr	*Utricularia intermedia*
UtrcMinr	*Utricularia minor*
VaccOxyc	*Vaccinium oxycoccos*
ViciCrac	*Vicia cracca*

Appendix 3. Confidence intervals of quantile regressions

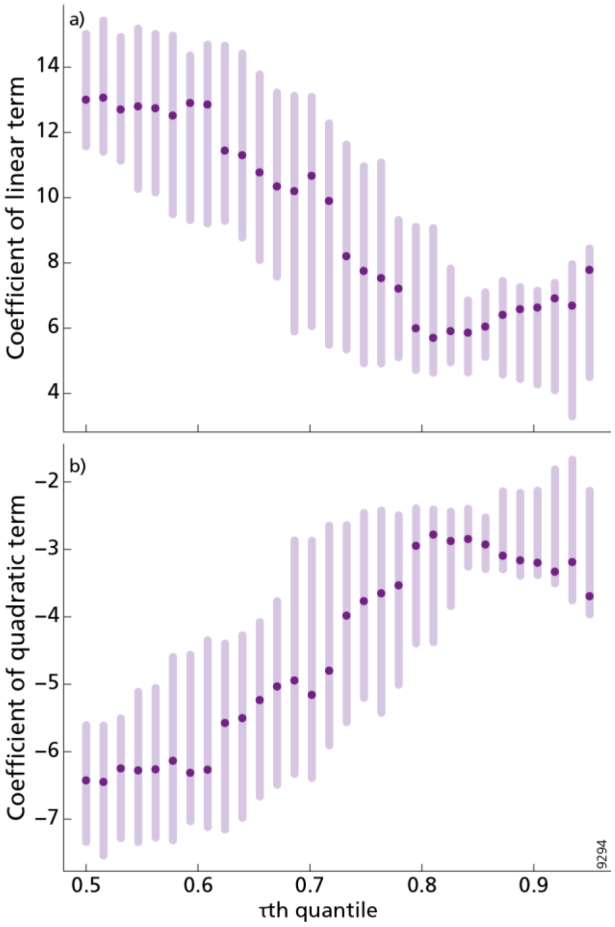

Figure S1. Ninety-five per cent confidence intervals of the quantile regression coefficients belonging to figure 3A. Estimated quantile regression coefficients (black dots), for a) the linear term and b) the quadratic term of the quadratic regression equation. In both panels, the grey bars indicate the 95% confidence interval around the fitted coefficient value (black dots). Note that niche position and niche width were both calculated using log(N:P), removing the need for further transformation of the variable.
